# Machine learning models in predicting health care costs in patients with a recent acute coronary syndrome: A prospective pilot study

**DOI:** 10.1016/j.cvdhj.2023.05.001

**Published:** 2023-05-13

**Authors:** Arto J. Hautala, Babooshka Shavazipour, Bekir Afsar, Mikko P. Tulppo, Kaisa Miettinen

**Affiliations:** ∗ Faculty of Sport and Health Sciences, University of Jyvaskyla, Jyvaskyla, Finland; † Faculty of Information Technology, University of Jyvaskyla, Jyvaskyla, Finland; ‡ Research Unit of Biomedicine and Internal Medicine, Medical Research Center Oulu, Oulu University Hospital, University of Oulu, Oulu, Finland

**Keywords:** Coronary artery disease, Coronary heart disease, Artificial intelligence, Health care costs, Economic evaluation

## Abstract

**Background:**

Health care budgets are limited, requiring the optimal use of resources. Machine learning (ML) methods may have an enormous potential for effective use of health care resources.

**Objective:**

We assessed the applicability of selected ML tools to evaluate the contribution of known risk markers for prognosis of coronary artery disease to predict health care costs for all reasons in patients with a recent acute coronary syndrome (n = 65, aged 65 ± 9 years) for 1-year follow-up.

**Methods:**

Risk markers were assessed at baseline, and health care costs were collected from electronic health registries. The Cross-decomposition algorithms were used to rank the considered risk markers based on their impacts on variances. Then regression analysis was performed to predict costs by entering the first top-ranking risk marker and adding the next-best markers, one by one, to build up altogether 13 predictive models.

**Results:**

The average annual health care costs were €2601 ± €5378 per patient. The Depression Scale showed the highest predictive value (r = 0.395), accounting for 16% of the costs (*P* = .001). When the next 2 ranked markers (LDL cholesterol, r = 0.230; and left ventricular ejection fraction, r = -0.227, respectively) were added to the model, the predictive value was 24% for the costs (*P* = .001).

**Conclusion:**

Higher depression score is the primary variable forecasting health care costs in 1-year follow-up among acute coronary syndrome patients. The ML tools may help decision-making when planning optimal utilization of treatment strategies.

**Key Findings**

• Advanced data analytics and machine learning tools can potentially be used to predict health care costs in real-world clinical settings.
• Our pilot study showed for the first time, using machine learning tools, that depression, expressed as higher depression scores, is the primary health measurement forecasting health care costs for all reasons, followed by low-density lipoprotein cholesterol and left ventricular ejection fraction, in 1-year follow-up among acute coronary syndrome patients.
• Applications of machine learning and artificial intelligence methods to health care costs can provide information that may be helpful for decision-making when planning optimal utilization of treatment strategies and resources in health care settings.

## Introduction

Cardiovascular disease incidence and mortality rates are declining in many countries in Europe but still remain a major cause of morbidity and mortality^1^ with a significant impact on health care costs. The economic burden of cardiovascular diseases in the European Union region was evaluated to be €169 billion annually and 62% of these costs were related to health care.^2^ The data from the United States show that expenditure on cardiovascular disease and cardiovascular risk factors in 2016 was $320 billion. Health services for ischemic heart disease ($80 billion) and treatment of hypertension ($71 billion) were the main causes of the costs, followed by treatment of hyperlipidemia.^3^ Based on the EUROASPIRE survey,^4^ costs of optimized tailored prevention such as smoking cessation, diet and exercise, better management of elevated blood pressure and/or low-density lipoprotein (LDL) cholesterol, and savings of avoided events were estimated based on country-specific data. The results showed that optimizing secondary prevention is clearly cost-effective compared with the current general guideline–oriented prevention.^4^

Health care providers worldwide are required to set priorities and allocate resources within the constraint of limited funding. However, decision makers may not be well equipped to make explicit rationing decisions and may often rely on historical or political resource allocation processes.^5^ Therefore, economic evaluation of health care for operational planning and decision-making is vital for allocation of resources to effective treatments that provide patients the greatest possible health benefits at reasonable costs. Since health care systems, care practices, and relative prices of health care investments vary from country to country, it is important to have country-specific data to support decision-making.^6^

Machine learning (ML) and artificial intelligence methods may have a considerably high potential for both effective and cost-effective use of health care resources when implemented in clinical practice.^7^^,^^8^ For example, Schwalm and colleagues^9^ showed recently that an ML prediction model used as an online decision support tool by referring physicians could improve the diagnostic yield of invasive coronary angiography in stable coronary artery disease (CAD) patients. The decision-maker planning for optimal use of health care resources may benefit from the prediction model of the most important risk factor or combination of those contributing most to health care costs. The feature importance analysis is widely used in predictive modeling, representing the significance of the input features at the target variables prediction by calculating predictive scores.^10^ We conducted an analysis using selected feature importance analysis tools to assess the contribution of well-addressed causal and modifiable risk markers for prognosis of CAD at baseline to predict health care costs in patients with a recent acute coronary syndrome (ACS) for 1-year follow-up in the Finnish health care system.

## Methods

### Study population

This study is part of the EFEX-CARE (Effectiveness of Exercise Cardiac Rehabilitation) study that has been registered at ClinicalTrials.gov (Identifier Record NCT01916525). The patients in the EFEX-CARE study have been recruited from a consecutive series of ACS patients in the Division of Cardiology of the Oulu University Hospital. They all underwent coronary angiography to confirm the CAD. The study population of the EFEX-CARE study has been previously described in detail,^11^ but to put it briefly, exclusion criteria included NYHA class ≥III, scheduled or emergency procedure for bypass surgery, unstable angina pectoris, severe peripheral atherosclerosis, diabetic retinopathy or neuropathy, or inability of independent daily physical activity, eg, owing to musculoskeletal problems. In this study, we report health care costs for 1 year follow-up and risk marker data at baseline measured about 2–3 weeks after their hospital discharge for the patients treated according to usual care. Altogether, all data needed for analysis were available for 65 patients. The study reported in this paper adhered to the CONSORT guidelines and was carried out according to the Declaration of Helsinki; the local committee of research ethics of the Northern Ostrobothnia Hospital District approved the protocol. All the subjects gave written informed consent.

### Assessment of patient characteristics, risk markers, and health care costs

Body weight and height were measured to assess body composition. Blood pressure was measured in a supine position after a 10-minute resting period according to the current guideline. Self-rated depression was assessed by using the Depression Scale (DEPS) questionnaire.^12^ The hospital registry and standard questionnaires were used to gather the data regarding smoking status, alcohol use disorders identification (AUDIT-C),^13^ medication, history of acute myocardial infarction, and revascularization. Assessment of left ventricular systolic function was performed using 2-D echocardiography (Vivid 7; GE Healthcare, Wauwatosa, WI). Blood samples from fasting stage were obtained for analysis of plasma glucose and glycated hemoglobin (HbA1c), blood lipids, insulin, and high-sensitivity C-reactive protein after a 12-hour overnight fast using consistent methods (Oulu University Hospital, Oulu, Finland). An incremental symptom-limited maximal exercise test was performed at the Oulu University Hospital on a bicycle ergometer (Monark Ergomedic 839 E; Monark Exercise AB, Vansbro, Sweden) for assessment of maximal physical exercise capacity (metabolic equivalents). The 15D questionnaire was used to record health-related quality of life^14^ and it was completed by the patients at the hospital before hospital discharge. In the estimation of health care costs, both specialized and primary health care services, as well as the costs of occupational health care services, were considered. Social security ID numbers were used to determine visits for ambulatory care, number of treatment days, and use of external services to calculate health care costs arising from the use of health services on the part of specialized health care. The exact costs were measured based on invoicing (using Diagnosis Related Groups classification). Information on the use of primary health care and costs related to it was obtained from electronic health registries by using unique social security ID numbers to determine visits to the doctor, other significant examinations such as large radiographs, and in-ward treatment days. Furthermore, the use of home care and possible institutional care (eg, assisted care home, etc) was determined from registries. The report of the Social Insurance Institute of Finland (KELA)^15^ was used to estimate occupational health care service costs. Finally, all costs were managed as 2015 values. Because of the 1-year time horizon of the analysis, no discounting was applied.

### Development of predictive models

In predictive modeling, the significance of the input features at the target variable predictions is represented by some scores calculated through a so-called feature importance analysis.^10^ In other words, these scores demonstrate the importance of a feature/variable for a prediction. Indeed, feature importance analysis is often used, as feature selection, to reduce the number of input variables, both to reduce the computational cost of modeling and, in some cases, to improve the model’s performance. These feature importance scores provide insight into the data and models and play a crucial role in improving the efficiency of the predictive models through feature selection^16^^,^^17^ and dimensionality reduction.^18^^,^^19^ Various feature importance methods have been developed in the literature, eg, based on statistical correlations and variances. However, the choice of methods depends on variables and the type of data. Therefore, it is recommended to evaluate various techniques to find suitable ones.

We conducted a feature importance analysis on our dataset to check the significance of selected risk factors in predicting all health care costs. After we tested various feature importance methods, the following ones proved their stability in several tests on randomly selected subgroups of samples from the dataset: Cross decomposition^20^; partial least squares (PLS) canonical analysis (PLSC), PLS based on singular value decomposition (PLSSVD), PLS regression (PLSRegression), and canonical correlation analysis (CCA) algorithms rank the considered risk factors based on their impacts on variances (ie, show which risk factor leads to the highest variance in costs). PLSRegression ranks considered risk factors based on absolute values of the correlations between the risk factor and the costs. Analysis of variance (ANOVA) test has also been used for feature selection to rank considered risk factors based on their *P* values. The used methods reflect the intrinsic predictive value of the risk factors and are not dependent on a particular predictive model that makes them more suitable in our case. PLS estimators are particularly suited when there is multicollinearity among the risk factors.^21^

After ranking of risk factors for health care costs, a linear regression analysis was performed to predict costs by entering the first top-ranking risk marker and adding the next-best markers, one by one, to build up altogether 13 predictive models. Descriptive statistical analyses were conducted using means, standard deviations (SDs), and proportions, as appropriate. SPSS software (SPSS 26; SPSS Inc, Chicago, IL) was used for predictive data analyses. Statistical significance was defined as a *P* value <.05 for all tests.

## Results

Baseline demographics, clinical characteristics, and medication use of the study participants are illustrated in Table 1. The total average cost per ACS patient for all reasons was €2601 ± 5378 for a 1-year follow-up.

**Table 1:** Baseline demographics, clinical characteristics, health care costs, and medication use of the study group (n = 65)

Variable	ACS patients
Men	46 (71%)
Patients with T2D	11 (17%)
Age, years	65 ± 9
Weight, kg	83 ± 14
BMI, kg/m^2^	28.0 ± 4.3
Systolic BP, mm Hg	137 ± 22
Diastolic BP, mm Hg	78 ± 11
Maximal exercise capacity, MET	5.6 ± 1.7
Quality of life, 15D scale	0.90 ± 0.08
AUDIT-C for alcohol use	2.9 ± 2.4
Depression scale	4.6 ± 5.3
Current smoker	8 (12%)
Total average health care cost per patient	
Cost for all reasons (€)	2601 ± 5378
History of AMI	
NSTEMI	45 (51%)
STEMI	22 (34%)
Revascularization	
PCI	55 (85%)
Earlier CABG	8 (12%)
Cardiac function	
LVEF, %	62 ± 7
CCS class	1.6 ± 0.6
Laboratory analyses	
HbA1c, %	6.0 ± 0.8
Fasting plasma glucose, mmol/L	6.0 ± 1.0
Total cholesterol, mmol/L	3.8 ± 0.7
HDL cholesterol, mmol/L	1.2 ± 0.3
LDL cholesterol, mmol/L	2.1 ± 0.7
Triglycerides, mmol/L	1.3 ± 0.6
hs-CRP, mg/L	2.7 ± 6.0
Medication	
Beta-blockers	56 (86%)
ACEI or ARB	54 (83%)
Lipids	64 (98%)
Anticoagulants	64 (98%)
Calcium antagonists	17 (26%)
Nitrates	18 (28%)
Diuretics	15 (23%)

Values are means ± SD or number (percentage) of subjects.

15D = health-related quality of life questionnaire; ACEI = angiotensin-converting enzyme inhibitor; ACS = acute coronary syndrome; AMI = acute myocardial infarction; ARB = angiotensin receptor blocker; AUDIT-C = Alcohol Use Disorders Identification Test; BMI = body mass index; BP = blood pressure; CABG = coronary artery bypass graft; CCS = Canadian Cardiovascular Society grading of angina pectoris; HbA1c = glycated hemoglobin; HDL = high-density lipoprotein; hs-CRP = high-sensitivity C-reactive protein; LDL = low-density lipoprotein; LVEF = left ventricular ejection fraction; MET = metabolic equivalent; NSTEMI = non-ST-segment elevation myocardial infarction; PCI = percutaneous coronary intervention; STEMI = ST-segment elevation myocardial infarction; T2D = type 2 diabetes.

**Figure 1:**
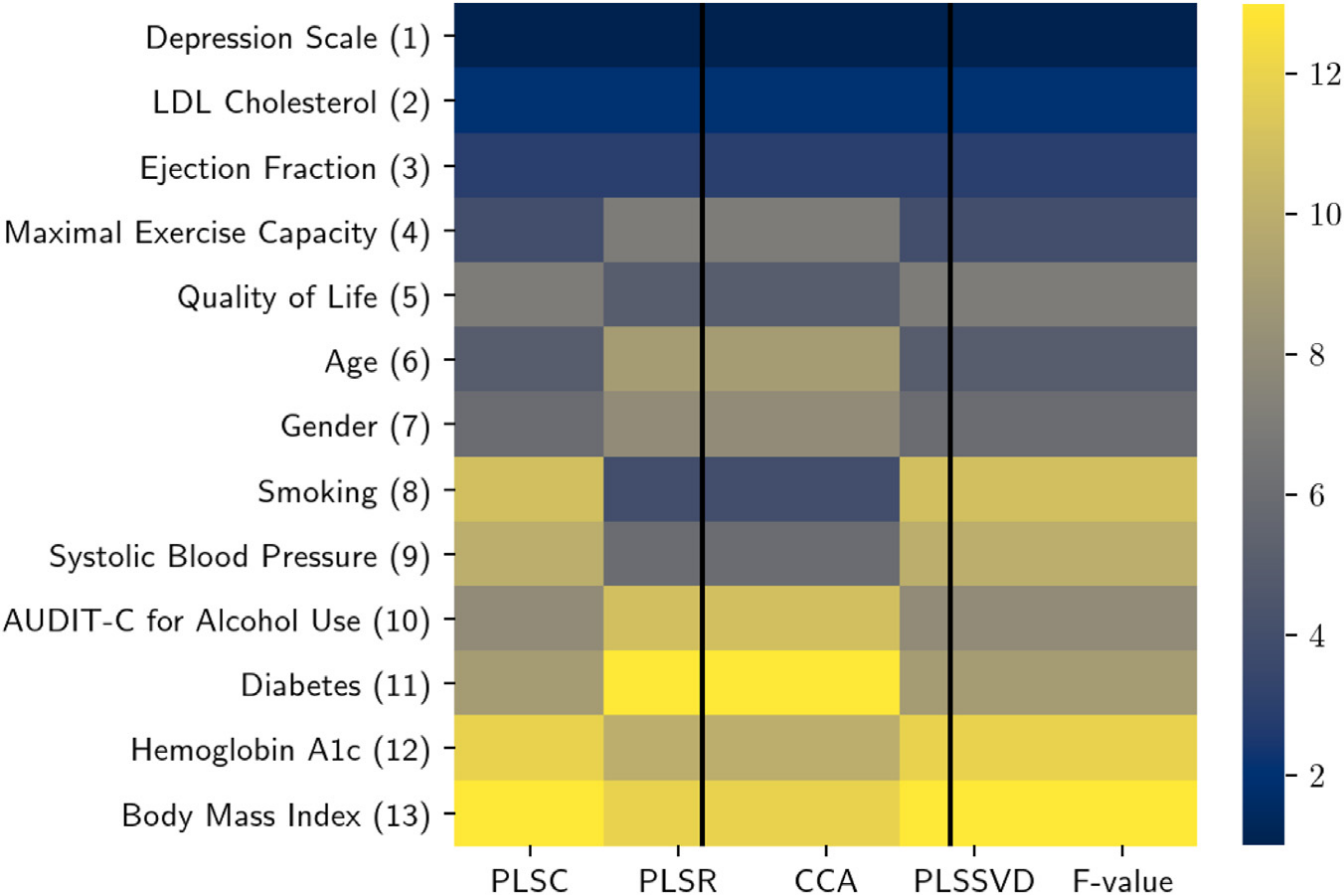
Rank aggregation of the risk factors calculated by different methods (each column represents a feature selection method). The lower rank value (darker color in the heatmap) denotes the higher importance of the risk factor. CCA = canonical correlation analysis; LDL = low-density lipoprotein; PLSC = partial least squares canonical analysis; PLSR = partial least squares regression; PLSSVD = partial least squares based on singular value decomposition; F value = value from the analysis of variance.

The ranking of risk factors for prediction of the health care cost is presented in Figure 1. The color code on the right side represents the risk factors ranking (1–13) in each feature selection method. The lower rank value (darker color in the heatmap) denotes the higher importance of the risk factor. The numbers in parentheses (1–13) show the aggregated rank of each risk factor over their ranking found in various methods.

Table 2 shows the final predictive models and their contributions to the costs. Furthermore, the direction of each risk marker contribution (negative or positive) is shown as correlation values. The DEPS showed the highest predictive value (r = 0.395), accounting for 16% of the costs (*P* = .001). Those patients who showed higher scores for depression had higher health care costs. When the next 2 ranked markers (LDL cholesterol, r = 0.230; and left ventricular ejection fraction [LVEF], r = -0.227, respectively) were added to the model, the predictive value was 24% for the costs (*P* = .001). Finally, having all 13 risk markers (including, eg, smoking, systolic blood pressure, and diabetes) in the model, they predicted 30% of the costs (*P* = .094).

**Table 2:** Linear regression analysis models for prediction of health care costs

Risk markers	R correlation	Model	R^2^	*P* value
Depression Scale	0.395	1	0.156	.001
LDL cholesterol	0.230	2	0.190	.001
Ejection fraction	-0.227	3	0.240	.001
Maximal exercise capacity	-0.142	4	0.245	.002
Quality of life, 15D	-0.106	5	0.251	.004
Age	0.092	6	0.273	.004
Sex	0.090	7	0.273	.008
Smoking	-0.089	8	0.274	.016
Systolic blood pressure	-0.070	9	0.276	.026
AUDIT-C for alcohol use	-0.050	10	0.285	.035
Diabetes	-0.048	11	0.298	.042
HbA1c	-0.027	12	0.299	.064
Body mass index	-0.022	13	0.300	.094

The models were defined according to ranking analysis for well-addressed causal and modifiable risk markers for prognosis of coronary artery disease at baseline. Model 1 includes top-ranking risk marker Depression Scale. Models from 2 to 13 were defined by entering the second-highest parameter (LDL cholesterol) to the model, then the next-highest risk markers were added one by one to the defined models (3, ejection fraction; 4, maximal exercise capacity; 5, quality of life; 6, age; 7, sex; 8, smoking; 9, systolic blood pressure; 10, AUDIT-C for alcohol use; 11, diabetes; 12, HbA1c; and 13, body mass index). Abbreviations as in Table 1.

## Discussion

The present study demonstrated that selected ML tools are applicable to predict health care costs for all reasons in 1-year follow-up when assessing the contribution of well-addressed causal and modifiable risk markers for prognosis of CAD collected at baseline in patients with a recent ACS. We found that depression expressed as the higher DEPS score is the primary contributing factor of health care costs in 1-year follow-up, followed by a higher LDL cholesterol level and lower values of LVEF. These results may be useful for decision-making when planning and focusing on optimal utilization of health care resources. Additionally, our findings may highlight the potential use of sophisticated ML data analytics tools in real-world clinical settings when making economic analyses to support decision-making.

At baseline, the most dominant predictors of all health care service costs in 1-year follow-up were related to a higher level of depression, a higher level of LDL cholesterol, a lower level of ejection fraction, a lower level of exercise capacity, and a lower level of health-related quality of life, accounting for about 25% of the costs in stable ACS patients treated according to the current guidelines. All those risk factors are well addressed as important causal and modifiable factors for the prognosis of disease.^22^ Interestingly, psychosocial risk factors, such as depression, have shown their importance in affecting cardiovascular prognosis, treatment adherence, quality of life, and sudden cardiac death.^23^^,^^24^ It is notable that in the present study, the highest level of correlation between health care costs and the depression score might be considered as a moderate association (r = 0.395), since a high level of correlation usually exceeds values of >0.5 and could be interpreted as a strong association. However, depressive symptoms have been shown to be strongly associated with higher levels of stress, low social support, unemployment, low family income, and unhealthy lifestyle such as low physical activity, low fruit and vegetable intake, and excessive salt consumption in CAD patients.^25^ Symptoms of depression are highly prevalent in stable CAD patients, and their long-term trajectories are suggested to be the single biggest driver of health care costs.^26^ Therefore, management of depression symptoms might be one of the primary focuses for policymakers and decision makers in planning treatment and resources for stable CAD patients.^23^

As mentioned above, depression is a common comorbidity in CAD patients and numerous potential mechanisms have been postulated for the relationship between depression and CAD. It has been documented that several clinical factors can be driving depression concurrently and thus may confound results when aiming to interpret and define a causal risk factor for depression. This kind of analysis may require evidence that reduction of the risk factor reduces risk.^27^^,^^28^ The DEPS scale we used in this study is a 10-item self-report scale that assesses the severity of depressive symptoms. Regarding the clinically meaningful value of the DEPS scale, it has been suggested that a score of 10 or higher on the DEPS scale is a useful cutoff for identifying clinically significant depressive symptoms.^29^ Furthermore, it is important to note that a DEPS score of 10 or higher should not be used as the sole basis for diagnosing depression. A comprehensive clinical evaluation, including a thorough history and physical examination, is necessary to make an accurate diagnosis and develop an appropriate treatment plan for depression.

Even though not in the scope of the present study, we performed further analysis using the DEPS score of 10 to find out if the measures we have assessed, including medication (presented in Table 1), are associated with the DEPS scale. Seven patients had a value of 10 or higher. The only parameter associated with the DEPS scale was quality of life, assessed with 15D questionnaire (r = -0.396, *P* = .001).

In the present study, the selected feature importance methods showed their applicability to rank well-known risk markers to find the most preferred first-order targets of risk markers to contribute to health care costs. We also used and evaluated some other relevant risk markers for the ACS population in the development process. For example, since high-sensitivity C-reactive protein has been shown to be an independent predictor for adverse cardiovascular events among CAD patients,^30^ we tested if it contributes to the order of leading predictive risk markers. We found that including high-sensitivity C-reactive protein in the analysis as an extra risk marker will not change the results. Furthermore, we assessed if the order of the leading predictive risk marker will change if we remove, one by one, the risk markers ranked from 6 to 13. Despite exclusion of the risk markers from the feature importance analysis, the order of the 5 leading markers remained the same. Therefore, we believe that in addition to selected feature importance analysis tools, the selected risk markers included were relevant and valid to the performed analysis.

The use of health care services in the present study was derived from hospital records instead of, for example, from patient self-reports, thereby eliminating recall bias. Secondly, the characteristics of patients at the baseline were widely assessed, including clinical status, medication, and comprehensive laboratory analysis. We feel that these are the strengths of this study. A limitation of this pilot study is that the patient sample in the EFEX-CARE study is small and may be partly selected, which could limit the generalizability to a broader population of ACS patients with significant comorbidities. We showed that after combining all the 13 studied markers, only 30% of the costs could be predicted by this model. This could be interpreted as a relatively low rate. However, the proprietary nature of economic data, and the fact that elements of health care costs are coming from different entities, may at least partly explain our results. For example, we were able to analyze direct health care costs, but not indirect costs such as the expenses incurred from the cessation or reduction of work productivity. The other question that remains open is whether it would be possible to raise the predictive value by adding more variables; this could be a target for future research. Additionally, although we carefully assessed various feature importance methods to prove their stability, the relatively low number of samples and multicollinearity among the risk factors may raise some caution in overall interpretation and generalizability in overall interpretation of the results. However, the proposed methodology is generic enough to be applied in any field or setting of medical and health care in which risk profiles of patients exist and health care costs for certain periods are assessed.

Because health care budgets are limited worldwide, there is a crucial need for strategies of health care systems that prove to be cost-effective. The need for care strategies should at the same time be low cost and give the best effect for care. However, direct assessment of costs is not reasonable in different countries because of differences in social and health care services nationally. Therefore, the results of the present study may be useful for policymakers especially in the Finnish health care system when planning and deciding how limited health care resources should be used in the optimal way.

## Conclusion

Our study showed that depression, expressed as higher depression scores, is the primary factor forecasting health care costs for all reasons, followed by LDL cholesterol and LVEF, in 1-year follow-up among ACS patients. These results are helpful for decision-making when planning optimal utilization of treatment strategies and resources in different health care settings. Furthermore, our findings confirm the potential use of advanced data analytics and ML tools in real-world clinical settings.
